# Physician participation in quality improvement work- interest and opportunity: a cross-sectional survey

**DOI:** 10.1186/s12875-022-01878-6

**Published:** 2022-10-25

**Authors:** Ellen Tveter Deilkås, Judith Rosta, Fredrik Baathe, Eirik Søfteland, Åse Stavland Lexberg, Olav Røise, Karin Isaksson Rø

**Affiliations:** 1 Health Services Research Unit, Akershus University Hospital, Lørenskog, Norway; 2 Institute for Studies of the Medical Profession (LEFO), Oslo, Norway; 3 The Institute of Stress Medicine, Region Västra Götaland, Gothenburg, Sweden; 4 Institute of Health and Care Sciences, Sahlgrenska Academy, Gothenburg University, Gothenburg, Sweden; 5 Department of Clinical Medicine, Faculty of Medicine, University of Bergen, Bergen, Norway; 6 Quality Department, Vestre Viken Hospital Trust, Drammen, Norway; 7 Institute of Clinical Medicine, Faculty of Medicine, University of Oslo, Oslo, Norway; 8 Faculty of Health Sciences, SHARE – Centre for Resilience in Healthcare, University of Stavanger, Stavanger, Norway

**Keywords:** Patient safety, Quality Improvement, Physicians, Work environment

## Abstract

**Background:**

Lack of physician involvement in quality improvement threatens the success and sustainability of quality improvement measures. It is therefore important to assess physicians´ interests and opportunities to be involved in quality improvement and their experiences of such participation, both in hospital and general practice.

**Methods:**

A cross-sectional postal survey was conducted on a representative sample of physicians in different job positions in Norway in 2019.

**Results:**

The response rate was 72.6% (1513 of 2085). A large proportion (85.7%) of the physicians wanted to participate in quality improvement, and 68.6% had actively done so in the last year. Physicians’ interest in quality improvement and their active participation was significantly related to the designated time for quality improvement in their work-hour schedule (p < 0.001). Only 16.7% reported time designated for quality improvement in their own work hours. When time was designated, 86.6% of the physicians reported participation in quality improvement, compared to 63.7% when time was not specially designated.

**Conclusions:**

This study shows that physicians want to participate in quality improvement, but only a few have designated time to allow continuous involvement. Physicians with designated time participate significantly more. Future quality programs should involve physicians more actively by explicitly designating their time to participate in quality improvement work. We need further studies to explore why managers do not facilitate physicians´ participation in quality improvement.

**Supplementary Information:**

The online version contains supplementary material available at 10.1186/s12875-022-01878-6.

## Background

Lack of physician involvement in quality improvement work (QIW) is acknowledged as a prevalent problem for the success and sustainability of quality improvement (QI) measures [1–4]. QIW is complex and depends on structures and processes, cooperation between multiple professions, power distance and the cultures and values of an organization [5–11]. QIW also requires the active participation of all healthcare professionals with contextual insight into the workplace that is to be improved [9, 12]. The American Institute of Medicine (IOM) has defined quality as the extent to which healthcare is safe, effective, involves users, is continuous and coordinated, efficient, and fairly distributed [13]. Norwegian legislation adopted the IOM quality dimensions in 2016 and in fact required healthcare leaders to implement QI measures according to the Plan-Do-Study-Act (PDSA) quality cycle [14]. The PDSA cycle implies repeated evaluation and adjustments to achieve the intended effect [14–16]. Despite relevant legislation and more practicing physicians and nurses per 1000 inhabitants, Norway still has challenges with quality improvement work compared to other countries in the Organization for Economic Co-operation and Development (OECD) [17].

Several studies have explored the lack of involvement of physicians in QIW [18]. Traditional physician culture and professional fulfillment have focused on research on providing good patient care and research on new medical treatments rather than participation in QIW [1, 19, 20]. Of course, QIW also aims to provide good patient care, but with a wider, organizational perspective, whereas physicians often feel a responsibility and engagement in the individual patient and research rather than for system quality [20]. In addition, physicians report that management seldom recognizes their provision of high-quality patient care and rarely includes them in QIW processes and systems development [21].

Strong healthcare leadership from the top promotes clinical physicians’ involvement in QIW [22]. Dual lines of authority found in many hospitals require visible commitment to QIW from both senior managers and physician leaders, as well as from strong actively engaged boards [22]. To encourage physicians to initiate and participate in clinical quality improvement projects, the key is to involve the hospital’s own physician leaders in strategic planning, policymaking, and other governance activities [22].

However, managerial priority for safe and effective care has declined, to meet quantitative production targets and budget constraints [21]. In a recent study, significantly fewer physicians compared to nurses experienced improvement measurements in their ward as relevant or that they had been informed about how the measurements were chosen, and if the measures were used for evaluation of improvement. Physicians also participated less frequently than nurses in the risk board meetings [23]. Although several previous studies explore the lack of physician involvement in QIW, few studies have actually investigated the prevalence of physicians reporting active participation in QIW at their workplace or their reported interest in participating.

This study is based on the survey and investigates physicians´ interest in participation and their actual participation in QIW and how this is influenced by organized opportunities in the organization (including designated time). Our hypothesis is that physicians’ opportunities to carry out QIW promotes their participation.

Young physicians might need extra motivation for QIW. A study found an inherent engagement for developing and improving clinical skills among young doctors and that this knowledge spreads in the networks between younger doctors. But there was no indication of a corresponding engagement or network for QIW [24].

Time for QIW can also depend on the physician´s work-life balance, where young physicians, and especially women, have a higher risk of experiencing work-home interface stress [25]. This can interfere with the possibility of designating time for QIW [24]. Fewer younger than older hospital physicians experience that their input is valued by the hospital leadership [26]. Consequently, it is reasonable to think that younger physicians experience fewer opportunities to improve their work processes than older physicians do, and it is therefore important to assess their opportunities to participate in QIW.

The workplace can also influence participation in QIW. Most of the General Practitioners (GPs) in Norway are in private practice and thus in the position of being employers themselves, in contrast to hospital doctors who are employees. GPs tend to organize themselves in groups that share premises, and have more or less formalized agreements about sharing responsibilities and costs, also for employees. Clinical leaders and management in general have a special responsibility to promote and facilitate QIW [22] As employers, this special responsibility applies also for GPs in private practice. At the same time, these companies are often very small, and the GPs have during the last 10 years had a marked increase in amount of work tasks [27] and a marked increase in perceived stress [28]. It is therefore important to study how GPs relate to QIW, compared with both managers and other physicians in Norway.

## Aims

1. The first aim of our study was to determine the prevalence of physicians reporting interest and actual active participation in QIW at their workplace.
2. The second aim of our study was to explore physicians’ opportunities and designated time to carry out QIW and to test the hypothesis that opportunities promote their participation.
3. The third aim of our study was to compare how different groups of physicians; especially GPs and young physicians report interest in and opportunities to, participate in QIW.

## Methods

### Design, setting and participants

Since 1994, The Institute for Studies of the Medical Profession (LEFO) in Norway has, approximately every second year, surveyed a representative sample (panel) of 1500 to 2200 active physicians with postal questionnaires about their health, quality of life and working conditions. Physicians that have agreed to take part in these surveys are called the physician panel. When necessary new, young doctors are invited to join, to compensate for doctors leaving the panel due to i.e. retirement. The panel is kept representative for Norwegian doctors in relation to gender, age and if they work in- or outside hospital. In this way the study sample represents an unbalanced cohort where the sample’s representative nature is maintained at all times [29] (Table 1). This study is based on survey data collected from November 2018 to April 2019.

**Table 1:** Sample, number of respondents, response rates and job positions for respondents for whom we have data on gender and age (< 70 years) in 2018–2019

	**2018–2019**
Sample, n	2 085
Respondents, n	1 513
Response rate, %	72.6
Job positions	n (females %)
All^a^	1 289 (53.9)
Doctors in hospital management^b^ and doctors in administrative position^c^	110 (33.6)
Senior hospital consultants	379 (50.4)
Specialty registrars	370 (71.9)
GPs and community medical officers^d^	306 (48.7)
Specialists in private practice	56 (33.9)
Doctors in academia^d^	68 (48.5)

^a^Interns (*n* = 17), doctors in other job positions (*n* = 77) and missing data (*n* = 129) were excluded

^b^Medical superintendent, head of department, chief senior consultant, head of the unit, senior consultant, head of section

^c^District medical officer, senior district medical officer, nursing home medical officer, visiting medical officer, physician at infant welfare clinic

^d^Professor, associate professor, research fellow, researcher

Four additional items regarding physicians’ interest, active participation and time allocated to participate in quality improvement work were included (Table 2). The items were developed in cooperation between the Institute for Studies of the Medical Profession (LEFO) and the board of the Physicians’ Association for Quality Improvement and Patient Safety (PAP).

**Table 2:** Four items in the panel questionnaire used to measure the main outcomes (in brackets)

1	Are you interested in participating in quality improvement work at your workplace? The response categories were yes and no. (**Expressed interest**)
2	My workplace is organised well to facilitate participation in quality improvement work. The response categories were from 1 "completely disagree" to 5 "completely agree". (**Opportunity**)^a^
3	Time is designated in my work-hour schedule to work with quality improvement. The response categories were from 1 "completely disagree" to 5 "completely agree". (**Opportunity**)^a^
4	Did you participate actively in quality improvement work during the last year at your work place? The response categories were yes and no. (**Active participation**)

^a^For the logistic regression analyses, answers were dichotomized into 0 (response categories from 1 to 3) and 1 (response categories 4 and 5)

### Main outcome measurements

The main outcome measures were physicians’ interest in, opportunities for, and active participation in QIW. The outcomes were measured using four items (Table 2). The response categories of the second and third items were on a five-point scale ranging from 1 ("completely disagree") to 5 ("completely agree"). To analyze the percentage of respondents who agreed, response categories were again dichotomized into 0 (response categories 1–3) and 1 (response categories 4–5)). QIW was defined as follows in line with the IOM and Norwegian legislation: Quality improvement work is about improving how patient treatment is organized and implemented so that it becomes more effective, safer, more user-friendly, and characterized by better coordination and continuity and more resource-effective. A common way to do this is to use the quality circle (PDSA), with repeated assessment of the effect and possible adjustment of quality improvement measures [13, 14].

### Independent variables

Outcome variables were analyzed according to the following independent variables: main job position (Table 1), gender and age.

### Analyses

Categorical data are presented with proportions. Chi square tests were performed to assess the relationships between having designated time for participation in QIW and interest in doing such work and actually participating in such work. The correlation between interest in and active participation in QIW was tested with Pearson’s correlation test.

Four logistic regression models were used to assess the simultaneous effect of gender, age and job positions on expressed interest, opportunity, and active participation in QIW. Respondents with missing data were excluded. Physicians with data on gender, age (< 70 years) and job positions were included.

The data were analysed using the IBM SPSS (Statistical Product and Service Solution) statistics software, version 26.

## Results

The response rate was 72.6% (1513 of 2085) (Table 1). The distribution of our sample was representative of practicing doctors in Norway in terms of age and gender and varied slightly regarding some job positions (hospital doctors, doctors in academia, interns, other positions), as described previously [28].

### Physicians’ interest in participating in QIW

85.7% of all the physicians expressed interest in participating in QIW (Table 3). Physicians´ interest in participating in QIW was highest for physicians in administrative positions (95.4%) and lowest for physicians in private practice (77.8%) (Table 3).

**Table 3:** Proportion of physicians who reported active participation and expressed interest in quality improvement work

	**Are you interested in participating in quality improvement work at your workplace?**		**Did you participate actively in quality improvement work during the last year at your work place?**	
	n	Yes (%)	n	Yes (%)
**All**	1274	85.7	1280	68.6
**Gender**				
Male	590	86.8	591	77.2
Female	684	84.8	689	61.2
**Age in years**				
≤ 35	392	83.2	392	53.3
36–45	329	82.1	329	64.7
46–55	240	90.4	242	81.0
56–69	313	89.1	317	82.0
**Job positions**				
Doctors in hospital management and in administrative position	109	95.4	110	94.5
Senior hospital consultants	372	86.0	376	75.0
Speciality registrars	368	80.7	368	48.1
General practitioners and Community medical officers	305	89.2	305	74.4
Specialists in private practice	54	77.8	55	80.0
Doctors in academia	66	86.4	66	66.7

Expressed interest was independent of gender and age. Hospital managers and doctors working administratively were significantly more interested than senior hospital consultants, specialty registrars and specialists in private practice (Table 4).

**Table 4:** Logistic regression analyses on physicians expressed interest in quality improvement work and active participation in quality improvement work as response variables (0 = no; 1 = yes)

	**Are you interested in participating in quality improvement work at your workplace? (*n* = 1274)**								**Did you participate actively in quality improvement work during the last year at your work place?** (*n* = 1280)							
	**Univariate analyses**				**Multivariate analyses**				**Univariate analyses**				**Multivariate analyses**			
	OR	95% CI Lower—Upper		Sig	OR	95% CI Lower—Upper		Sig	OR	95% CI Lower—Upper		Sig	OR	95% CI Lower—Upper		Sig
**Gender**																
Female	1				1				1				1			
Male	1.18	0.86	1.62	0.313	0.99	0.71	1.38	0.946	2.14	1.67	2.73	0.000	1.55	1.19	2.02	0.001
**Age in years**																
≤ 35	1				1				1				1			
36–45	0.93	0.63	1.36	0.699	0.78	0.49	1.23	0.279	1.61	1.19	2.17	0.002	0.98	0.68	1.40	0.907
46–55	1.91	1.15	3.16	0.012	1.47	0.78	2.76	0.230	3.73	2.56	5.44	0.000	1.65	1.03	2.65	0.039
56–69	1.66	1.07	2.59	0.025	1.25	0.69	2.24	0.466	3.99	2.82	5.66	0.000	1.58	1.01	2.50	0.047
**Job positions**																
Doctors in hospital management, doctors in administrative position	1				1				1				1			
Senior hospital consultants	0.29	0.12	0.76	0.011	0.32	0.12	0.83	0.019	0.17	0.07	0.41	0.000	0.20	0.09	0.47	0.000
Specialty registrars	0.20	0.08	0.51	0.001	0.25	0.09	0.68	0.007	0.05	0.02	0.13	0.000	0.09	0.04	0.22	0.000
GPs and community medical officers	0.39	0.15	1.04	0.061	0.43	0.16	1.15	0.093	0.17	0.07	0.40	0.000	0.20	0.08	0.48	0.000
Specialists in private practice	0.16	0.06	0.51	0.002	0.16	0.05	0.49	0.001	0.23	0.08	0.66	0.006	0.22	0.08	0.64	0.005
Doctors in academia	0.30	0.10	.952	0.041	0.34	0.11	1.08	0.068	0.12	0.04	0.30	0.000	0.14	0.05	0.38	0.000

### Physicians’ active participation in QIW

A total of 68.6% of all the physicians had actively participated in QIW the last year (Table 3). Prevalence of active participation in QIW was highest for physicians in administrative positions (94.5%) and lowest for specialty registrars (48.1%) (Table 3)

Prevalence of active participation was significantly higher among men than among women, and older doctors (≥ 46 years) compared to younger (≤ 35 years old), and significantly higher among physicians in administrative positions compared to all other job positions (Table 4). General Practitioners and community medical officers had a similar rate of active participation as senior hospital consultants and significantly lower than doctors in hospital management.

The correlation between physicians participating in QIW and expressing interest in participating in QIW was moderate and significant (Pearson 0.4 (medium correlation 0.30–0.49), p < 0.001). The discrepancy between younger physicians’ interest to do QIW and their active participation was much larger than for older physicians.

### Physicians’ perceptions of how the workplace facilitated participation in QIW

A total of 34.1% of physicians agreed that their workplace was organized well to facilitate participation in QIW, while only 16.7% agreed that time was designated for such work in their own work hours. Physicians in administrative positions agreed significantly more often that time was designated for QIW (33.9%) than senior hospital consultants, specialty registrars, GPs and Community medical officers, and specialists in private practice. Only 9% of specialty registrars agreed that time was designated for QIW in their own work hours (Table 5).

**Table 5:** Proportion of physicians agreeing that "My workplace is organized well to facilitate participation in quality improvement work" and “Time is designated in my work-hour schedule to work with quality improvement". The response categories were placed on a linear five-point scale from 1 ("completely disagree") to 5 ("completely agree"). To analyse the percentage of respondents who agreed, response categories were dichotomized into 0 (response categories 1–3) and 1 (response categories 4–5 (agreed))

	**My workplace is organized well to facilitate participation in quality improvement work**			**Time is designated in my work-hour schedule to work with quality improvement**		
		Categories 1–3	Categories 4–5		Categories 1–3	Categories 4–5
	n	%	%	n		%
**All**	1274	65.9	34.1	1275	83.3	16.7
**Gender**						
Male	588	58.2	41.8	589	79.5	20.5
Female	686	72.6	27.4	686	86.6	13.4
**Age in years**						
≤ 35	392	77.8	22.2	392	90.3	9.7
36–45	327	69.1	30.9	327	85.3	14.7
46–55	243	56.0	44.0	243	78.2	21.8
56–69	312	55.4	44.6	313	76.4	23.6
**Job positions**						
Doctors in hospital management and doctors in administrative position	109	36.7	63.3	109	66.1	33.9
Senior hospital consultants	374	67.6	32.4	375	82.7	17.3
Specialty registrars	366	77.0	23.0	366	91.0	9.0
GPs and community medical officers	304	67.4	32.6	304	83.9	16.1
Specialists in private practice	55	52.7	47.3	55	81.8	18.2
Doctors in academia	66	47.0	53.0	66	71.2	28.8

There were no significant gender differences, but significantly more of the oldest physicians (56–69) than the youngest physicians (≤ 35) agreed that they had designated time for quality improvement work in their work hours (Table 6).

**Table 6:** Logistic regression analyses on agreement with "My workplace is organized well to facilitate participation in quality improvement work" and "Time is designated in my work-hour schedule to work with quality improvement". The response categories were placed on a linear five-point scale from 1 ("completely disagree") to 5 ("completely agree"). To analyse the percentage of respondents who agreed, response categories were dichotomized into 0 (response categories 1–3) and 1 (response categories 4–5 (agreed))

	**My workplace is organized well to facilitate participation in quality improvement work** (*n* = 1 274)								**Time is designated in my work-hour schedule to work with quality improvement** (*n* = 1 275)							
	**Univariate analyses**				**Multivariate analyses**				**Univariate analyses**				**Multivariate analyses**			
	OR	95% CI Lower—Upper		Sig	OR	95% CI Lower—Upper		Sig	OR	95% CI Lower—Upper		Sig	OR	95% CI Lower—Upper		Sig
**Gender**																
Female	1				1				1				1			
Male	1.91	1.51	2.41	0.000	1.52	1.18	1.96	0.001	1.67	1.24	2.25	0.001	1.30	0.950	1.79	0.100
**Age in years**																
≤ 35	1				1				1				1			
36–45	1.57	1.12	2.19	0.008	1.42	0.96	2.10	0.083	1.60	1.02	2.52	0.042	1.24	0.731	2.11	0.423
46–55	2.76	1.95	3.91	0.000	2.22	1.40	3.51	0.001	2.60	1.65	4.09	0.000	1.74	0.971	3.13	0.063
56–69	2.82	2.03	3.90	0.000	2.05	1.32	3.20	0.002	2.88	1.89	4.41	0.000	1.84	1.044	3.25	0.035
**Job positions**																
Doctors in hospital management, doctors in administrative position	1				1				1				1			
Senior hospital consultants;	0.28	0.18	0.43	0.000	0.31	0.20	0.49	0.000	0.41	0.25	0.66	0.000	0.46	0.281	0.74	0.002
Specialty registrars;	0.17	0.11	0.27	0.000	0.35	0.20	0.62	0.000	0.19	0.11	0.33	0.000	0.34	0.171	0.66	0.002
GPs and community medical officers	0.28	0.18	0.44	0.000	0.35	0.22	0.56	0.000	0.38	0.23	0.62	0.000	0.44	0.265	0.74	0.002
Specialists in private practice	0.52	0.27	1.00	0.051	0.51	0.26	0.98	0.045	0.43	0.20	0.95	0.038	0.43	0.192	0.94	0.035
Doctors in academia	0.66	0.35	1.22	0.181	0.87	0.46	1.65	0.665	0.79	0.41	1.53	0.479	0.97	0.492	1.93	0.937

### Opportunity, interest and active participation in QIW

Among physicians with designated time, 91,8% expressed interest in doing quality improvement work compared to 83.6% among those without designated time (Chi^2^ = 10.2, *p* = 0.001). Among physicians with designated time, 86.6% had participated in QIW compared to 63.7% among those without designated time (Chi^2^ = 45.9, *p* < 0.001).

## Discussion

### Main findings

A very large proportion (85.7%) of all the physicians in our study wanted to participate in QIW, and another large proportion of physicians (68.6%) had already done so during the last year. Physicians’ interest in participating in quality improvement work and their active participation was moderately related to whether they had designated time for this in their work-hour schedule.

### Comparison with other studies

Our main study findings that physicians in general are positive about engaging in QIW contrasts with a general impression from previous studies that physicians do not participate in QIW [1]. Several explanations have been provided to explain the lack of physician participation, including lack of knowledge and harbouring attitudes that such knowledge is not part of the professional identity, as well as lack of managerial facilitations [30]. Recent qualitative studies show that physicians understand the importance of QIW in order to provide good quality patient care and secure a sustainable work environment that ensures professional fulfilment [19, 22]. However, few studies have quantified to what extent physicians want to participate in QIW. An exception is a US national physician survey from 2003, where 34% of physicians had participated in QIW [31]. The results in this study quantitatively reinforces the knowledge gained in the qualitative work. A large proportion of the physicians want to participate in QIW and report participating to a higher degree when they have designated time to do so. This underlines the importance of time management by managers and leaders to facilitate physician participation in QIW.

In parallel to formalized QIW projects many physicians are engaged in enhancing quality of patient care, for example through systematic registers of treatment outcomes for specific diagnoses. They report that they often experience a lack of interest from management in this work. Thus, there are measures of quality improvement, that physicians are engaged in, but that are not usually viewed as so-called "quality improvement work" [21]. Management involvement of physicians in defining important goals for QIW is therefore important. The percentage of physicians who had participated in QIW the last year (total of 69%) varied between 95% for physicians in administrative positions and 48% for specialty registrars. The percentage of physicians reporting designated working time for QIW varied between 34 and 9% for the same two groups. The fact that physicians with designated time had participated in QIW to a larger degree resonates with Donabedians’ structure, process and outcome framework and indicates that opportunity promotes participation in QIW [5]. Processes in health care depend on structures, such as time allocation, so that activities such as QIW may take place and give results [23].

### Explanations of physicians’ participation in QIW

Our study demonstrates a gap between a large proportion of physicians being interested in quality improvement work and a lower proportion of physicians actually taking part in such work. We also found a statistically significant but moderate relation between interest, and active participation in QIW and if they had designated time in their work schedule. A possible explanation may be that some physicians lack autonomy and work time to participate in QIW. The explanation is supported by a different study which shows that few Norwegian physicians experience opportunity to influence decisions that impact their working situation, autonomy to plan their own working time, or work tasks [26]. A later follow up study showed that perceived autonomy was lowest amongst junior physicians and females [32]. This matches the discrepancy between interest to do QIW and active participation, which was much larger for younger physicians’ than older physicians (Table 4).

Several conditions can contribute to Norwegian physicians’ lack of autonomy to participate in QIW. Heavy and stressful clinical work with many work hours and concurrency conflicts may potentially obstruct engagement in QIW [33]. Many physicians depend on their local leaders for the opportunity to participate in QIW without negatively affecting immediate patient care [19, 20, 34]. However, some leaders seem to think that QIW at the front line occurs by itself without careful ongoing systematic effort of clinical staff. Many workplaces do not recognize the need for support nor offer support to physicians who wish to engage in QIW [2]. Although there is legislation about the necessity of QIW processes, physicians in clinical work lacked the same opportunity to do QIW, as physicians in administrative positions have. Norwegian legislation unfortunately does not specify or demand structures that provide the capacity to involve all personnel groups and make QIW happen [35].

Not involving clinical physicians is problematic since QIW requires the participation of those with contextual knowledge [9]. Leaders who motivate and plan (by dedicating time) so that physicians can contribute can stimulate increased QIW participation of physicians. This is especially important for physicians in clinical positions (specialty registrars and senior hospital consultants) who depend on management to allocate time and have little opportunity to prioritize it themselves. This was experienced in one Norwegian medical division, which established "Improvement physicians" as a job position in 2012. "Improvement physicians" are specialty registrars with 20–50% of their work time designated to do QIW [36].

It is notable that the GPs interest in and participation in QIW so closely resembles that of senior consultants – and not hospital managers. Although GPs are mostly private practitioners, and thus in a manager position, their work situation probably resembles that of a senior consultant more than that of a manager. We need qualitative data to explore the factors that promote or restrict QIW in these groups.

Provided designated time to do QIW, physician engagement in QIW may reduce distractions and concurrency conflicts that cause stress and burnout and compromise professional performance [28, 33, 37]. QIW involvement may also improve such working conditions, the performance and professional fulfillment of physicians and probably other staff [38]. By teaching QIW skills to medical students and letting them engage in QIW during their studies, physician engagement may be promoted as well. Continuing medical education with networks for residents focusing specifically on QIW has also been found to promote participation in QIW in the clinic [39].

In addition to dedicated time, it is also important to engage physicians in a discussion about which quality of care measures that physicians find important to monitor and improve. For physicians and managers to both acknowledge the need for quality improvement in specific individual patient care, and to develop a more holistic and interdisciplinary view of quality with its organizational consequences for the department/hospital.

### Strengths and limitations

A major strength of this study is that it is based on data from a panel of physicians representative of a whole country with a response rate above 72%. QIW was explicitly defined in the questionnaire, reducing the risk of misinterpretations of the concepts meaning. A weakness of the study is that different groups of physicians might have different incentives to report their interest in QIW. Physicians in administrative positions who are more responsible for promoting and facilitating QIW would be expected to answer more positively [22]. A second weakness may be that we had not elaborated the qualitative meaning of "My workplace is organized well to facilitate participation in QIW". It would therefore seem that doctors could evaluate the workplace as well organized for QIW (approximately one-third of the doctors), with only 17% of the physicians reporting designated time to participate in such work.

## Conclusions

Our study demonstrates that physicians with designated work time for QIW were more likely to be interested and to have participated actively in QIW. As our study did not test an intervention, we cannot claim a causal relation. For future research, we recommend to follow up with a qualitative study, to uncover obstacles for physicians’ participation in QIW, and an intervention study to test the effect designated work time on physicians’ participation in QIW.

## Supplementary Information

**Additional file 1. **

## Data Availability

The datasets generated and/or analyzed during the current study are not publicly available. The dataset is part of a comprehensive longitudinal follow-up study of Norwegian doctors, which started in 1993. Respondents have consented to publish aggregated data, but not to openly publish data for individuals. Data are available from the corresponding author on reasonable request.

## Abbreviations

QIW: Quality Improvement Work
QI: Quality Improvement
PDSA: Plan-Do-Study-Act
IOM: The American Institute of Medicine
OECD: Organization for Economic Co-operation and Development
GP: General Practitioner
